# Gene therapy for PIDs: Progress, pitfalls and prospects

**DOI:** 10.1016/j.gene.2013.03.098

**Published:** 2013-08-10

**Authors:** Sayandip Mukherjee, Adrian J. Thrasher

**Affiliations:** a Centre for Immunodeficiency, Molecular Immunology Unit, University College London Institute of Child Health, 30 Guilford Street, London WC1N1EH, UK; b Great Ormond Street Hospital for Children, National Health Service Trust, London WC1N1EH, UK

**Keywords:** PIDs, primary immunodeficiency disorders, HSCs, haematopoietic stem cells, ADA-SCID, adenosine deaminase deficiency-severe combined immunodeficiency, SCID-X1, X-linked severe combined immunodeficiency, CGD, chronic granulomatous disorder, WAS, Wiskott–Aldrich syndrome, HSCT, haematopoietic stem cell transplant, HLA, human leukocyte antigen, SAE, serious adverse event, LTR, long terminal repeat, SIN, self-inactivating, ERT, enzyme replacement therapy, PEG, polyethylene glycol, IL, interleukin, MLV, murine leukaemia virus, GALV, gibbon ape leukaemia virus, GvHD, graft versus host disease, T-ALL, T cell-acute lymphoblastic leukaemia, NK cells, natural killer cells, NADPH, nicotinamide adenine dinucleotide phosphate hydrogen, IFN, interferon, ROS, reactive oxygen species, SFFV, spleen focus-forming virus, MDS, myelodysplastic syndrome, WASp, Wiskott–Aldrich syndrome protein, XLT, X-linked thrombocytopenia, XLN, X-linked neutropenia, VSV-G, vesicular stomatitis virus-G protein, LVs, lentiviral vectors, RVs, retroviral vectors, PGK, phosphoglycerokinase, EF-1α, elongation factor 1α, UCOE, ubiquitous chromatin opening element, miR, microRNA, LCR, locus control region, MN, meganucleases, ZFN, zinc finger nuclease, TALEN, transcription activator-like effector nucleases, HR, homologous recombination, DSB, double strand break, GSH, genomic safe harbour, PID, Gene therapy, HSCT, SCID, CGD, WAS

## Abstract

Substantial progress has been made in the past decade in treating several primary immunodeficiency disorders (PIDs) with gene therapy. Current approaches are based on ex-vivo transfer of therapeutic transgene via viral vectors to patient-derived autologous hematopoietic stem cells (HSCs) followed by transplantation back to the patient with or without conditioning. The overall outcome from all the clinical trials targeting different PIDs has been extremely encouraging but not without caveats. Malignant outcomes from insertional mutagenesis have featured prominently in the adverse events associated with these trials and have warranted intense pre-clinical investigation into defining the tendencies of different viral vectors for genomic integration. Coupled with issues pertaining to transgene expression, the therapeutic landscape has undergone a paradigm shift in determining safety, stability and efficacy of gene therapy approaches. In this review, we aim to summarize the progress made in the gene therapy trials targeting ADA-SCID, SCID-X1, CGD and WAS, review the pitfalls, and outline the recent advancements which are expected to further enhance favourable risk benefit ratios for gene therapeutic approaches in the future.

## Highlights

• PID gene therapy is a promising alternative to HSCT in absence of suitable donors.
• Gene therapy clinical trials targeting various PIDs have shown significant efficacy.
• Retroviral and lentiviral vectors are presently the mainstay of gene delivery.
• Insertional mutagenesis and vector silencing constitute the main concerns.
• Novel gene delivery approaches are currently being investigated and validated.

## 1 Introduction

Primary immunodeficiencies (PIDs) constitute a large (more than 300 gene mutations) and heterogeneous group of rare heritable disorders resulting in an underdeveloped and/or functionally compromised immune system. World-wide, the incidence of PIDs varies greatly from 1 in 600 to 1 in 500,000 live new-borns, depending both upon the specific disorder and the ethnicity of the population under study. Patients with PIDs display phenotypes which can range from being asymptomatic to manifestation of life-threatening conditions (e.g. various forms of severe combined immunodeficiency, SCID). Additionally, they might also suffer from auto-immune disorders and exhibit predisposition towards lympho-reticular malignancies (Kildebeck et al., 2012; Rivat et al., 2012).

In the clinic, allogeneic hematopoietic stem cell transplant (HSCT) from a human leukocyte antigen (HLA) matched donor confers significant therapeutic benefit to the patient with a success rate of more than 90%. Unfortunately, scarcity of HLA-matched donors (available for only one-third of patients) results in considerable reduction of successful engraftment with concomitant increases in morbidity and mortality. For this significant number of patients without a suitable donor, autologous transplantation of genetically corrected haematopoietic stem and progenitor cells offers a life-saving alternative (Qasim et al., 2009).

Major therapeutic benefits via gene transfer to HSCs was first demonstrated in SCID patients more than 10 years ago, and this type of protocol continues to exhibit promise with applications now including other haematological (such as Wiskott–Aldrich syndrome (WAS) and beta-thalassemia) and metabolic disorders (for e.g. X-linked adrenoleukodystrophy and metachromatic leukodystrophy). Most PIDs display Mendelian inheritance and therefore the introduction of a wild-type copy of the mutated gene into the stem cells of the patient should theoretically correct the disease phenotype. Further, it has been observed that for some PIDs including those involving T-cell immunodeficiencies, the gene-corrected HSC population exhibits a selective growth advantage in repopulating the bone-marrow (Antoine et al., 2003). Long-term data collected from clinical trials of PID patients treated with gene therapy (the majority of these trials have employed conventional γ-retroviral vectors) have shown clear signs of efficacy with more than 90% of the patients surviving without any serious adverse event (SAE). Considering the scenario of HLA-mismatch, these results are impressive when compared to the results from other alternative therapies (Booth et al., 2011). It is worthwhile to note that insertional mutagenesis-induced toxicity observed in approximately 10% of total patients shares a common mechanistic pathway i.e. upregulation of proto-oncogene expression stimulated by the proximity and presence of enhancer sequences within the U3 region of the 5′ long terminal repeat (LTR) of the γ-retroviral vectors used in these trials. To address this, the γ-retroviral vectors are being increasingly replaced by self-inactivating (SIN) retroviral vectors, which in pre-clinical studies have shown similar efficacy in terms of sustainable transgene expression, but reduced tendency for harmful mutagenesis (Seymour and Thrasher, 2012).

Based on the favourable risk benefit ratio observed in PID patients receiving gene therapy, it can be strongly argued that gene therapy has successfully graduated from the proof-of-principle stage and promises to be incorporated into mainstream medicine to prevent, alleviate, and provide long-term treatment for a wide variety of genetic disorders in situations where conventional therapies have failed or are unavailable. In this review, we aim to provide an overview of the gene therapy clinical trials conducted so far targeting the PIDs ADA-SCID, SCID-X1, CGD and WAS, discuss the advances in vector design and other gene targeting technologies in relation to the present pitfalls as witnessed in these trials, and finally reflect on the future of gene therapy in becoming a “game-changer” of 21st century medicine for rare or common, inherited or acquired genetic disorders.

## 2 Progress in PID gene therapy clinical trials

### 2.1 ADA-SCID

Under normal physiological circumstances, DNA and RNA are broken down inside the cell into toxic metabolites called deoxyadenosine and adenosine respectively, which when acted upon by the enzyme adenosine deaminase (ADA), are converted into the corresponding less toxic deoxyinosine and inosine as an essential step of the purine salvage pathway. ADA deficiency results in the accumulation of toxic metabolites in the intracellular as well as extracellular compartments causing impaired development of functional T, B and NK cells. This typically results in severe combined immunodeficiency (SCID) characterized by repeated and persistent infections from infancy which can be lethal without early clinical intervention. Additionally, since ADA is ubiquitously expressed in all tissues of the body, ADA-SCID patients suffer from a host of other abnormalities affecting the skeleton, GI tract, lung, liver and nervous system. Approximately 15% of the SCID patients worldwide suffer from ADA-SCID.

Allogeneic HSCT typically constitutes the treatment of choice for patients with a HLA-matched sibling or family donor (~ 88% success). However, the rates of success fall sharply when employing a matched-unrelated donor (~ 67%) or a haplo-identical sibling (~ 43%). Enzyme replacement therapy (ERT) in the form of weekly or bi-weekly intramuscular injection of polyethylene glycol-conjugated ADA (PEG-ADA) has been found to be effective in rescuing the disease phenotype, but unfortunately cannot be offered to all patients worldwide due to the high costs involved. Besides, long-term administration of PEG-ADA has also been found to have only partial efficacy (Gaspar et al., 2009; Montiel-Equihua et al., 2012).

Historically, ADA deficiency was the first SCID condition for which a genetic and molecular cause was identified and eventually transplantation of ADA-SCID patients with γ-retrovirus mediated gene-corrected autologous HSCs (from bone marrow or umbilical cord blood) in the early 1990s constituted the first attempts of treating a PID with gene therapy. However, the therapeutic benefits of these trials could not easily be determined as the patients continued ERT alongside gene therapy thereby potentially nullifying the survival and growth advantage of gene-corrected lymphocyte precursors and progenies. More recent evidence from an animal model of ADA-deficiency on the contrary would suggest that this is not an important effect, and that a lack of pre-conditioning is a key factor (Carbonaro et al., 2012). Promisingly, no toxicity was observed in these pilot trials, and gene-marked cells were detected in peripheral blood circulation even ten years after treatment, although significantly below the threshold levels required for achieving therapeutic benefit (Aiuti et al., 2002; Blaese et al., 1995; Bordignon et al., 1995).

Taking cues from these earlier attempts, several modifications were incorporated at various stages of the protocol for future clinical trials for ADA-SCID gene therapy. Briefly, these are the following:

a. Ex-vivo culture of HSCs was stimulated with a cytokine cocktail which promoted expansion without losing the ‘stemness’ of the CD34^+^ population.
b. Higher transduction efficiency of HSCs with the retroviral vector was achieved by employing fibronectin which enhances virus-cell co-localization.
c. Engraftment of the infused corrected HSCs was enhanced by including a mild-preconditioning regime.

The highlights of the recent clinical trials conducted in Italy, the UK and the USA have been summarized in Table 1. Except for the recent-most trial initiated in 2012, all the trials conducted so far have employed γ-retroviral vectors driving expression of the ADA gene from the 5′ viral LTR. Mild non-myeloablative conditioning regimens have been used in all these trials to make sufficient space for the transduced ADA-HSCs, and multi-lineage reconstitution following persistent engraftment has been demonstrated to confer clinical benefits i.e. adequate metabolic detoxification to a significant number of patients. Notably, approximately 75% of patients treated with gene therapy have been taken off ERT. Again, extended follow-up of the clinical trials has revealed no SAE although intriguingly, integration hotspots have been identified in some patients. However, clonal dominance and expansion of potentially dangerous clones leading to adverse physiological response have not been reported so far (Aiuti and Roncarolo, 2009; Aiuti et al., 2007, 2009; Candotti et al., 2012; Gaspar et al., 2011b).

To summarize (Table 1), the survival rate from ADA-SCID gene therapy has been 100% with efficacy comparing favourably with HSCT with fully matched donor and faring better than ERT in terms of costs involved and long-term immune reconstitution which includes correction of T regulatory cell function and B cell tolerance defects. Additionally, children's growth and bone age improved following treatment although were not normalized in all patients (Cavazzana-Calvo et al., 2012).

### 2.2 SCID-X1

X-linked SCID, accounting for 40%–50% of SCID cases reported world-wide, is caused by mutations in the IL2RG gene leading to defective expression of the common gamma chain (γc), a subunit shared by a host of cytokine receptors including interleukin (IL)-2, 4, 7, 9, 15 and 21 receptor complexes which play a vital role in lymphocyte development and function. SCID-X1 patients present profound immunological defects caused by low numbers or complete absence of T and NK cells, and presence of non-functional B cells (Fischer and Cavazzana-Calvo, 2008). Although allogeneic transplantation from HLA-identical donor has a high success rate, persistent defects in humoral or cellular functions have been reported for some patients resulting in partial immune recovery, autoimmunity and/or retarded growth (Neven et al., 2009).

The observation that spontaneous reversion of γc mutation was able to restore immunological competence indicated that lymphocyte progenitors with functional IL2RG gene possess selective advantage over the deficient progenitors, making SCID-X1 a strong candidate for gene therapy (Bousso et al., 2000). The first clinical gene therapy protocol conducted at Hôpital Necker (Paris, France) with nine patients was carried out by ex vivo γc gene transfer into autologous CD34^+^ cells using a conventional amphotropic murine leukaemia virus (MLV)-based vector in which IL2RG gene expression was driven by the viral LTR (Hacein-Bey-Abina et al., 2002). An equivalent study was performed on ten patients at Great Ormond Street Hospital (London, UK) with the exception of employing a gibbon ape leukaemia virus (GALV) envelope-pseudotyped MLV-based vector (Gaspar et al., 2004). Both trials omitted pre-conditioning of the patients before gene therapy. Taken together, 17 out of 19 patients who enrolled in these trials have demonstrated positive clinical outcome in terms of T cell recovery. For participants in both trials, long term follow-up studies have confirmed active thymopoiesis with a polyclonal and functional T cell receptor repertoire in the majority of patients. Restoration of humoral immunity was found to be partial but sufficient to withdraw some patients from immunoglobin replacement therapy. Interestingly, although cellular immune reconstitution is comparable to that of HLA-matched HSCT, molecular analysis has revealed a strong bias towards gene-marking in T and NK cells comparable to B cells and cells of the myeloid lineage. This proves that expression of γ chain confers a selective advantage to the T and NK cell progenitors albeit to a lesser degree for the NK cells in the absence of chemotherapy. Following on the success of Paris and London trials, SCID-X1 gene therapy was attempted in 5 older patients (age 10–20 years) but failed to show any significant clinical benefit most likely due to age-related factors and/or history of chronic infection and graft versus host disease (GVHD). This observation strengthens the recommendation that gene therapy should be attempted at the earliest opportunity after detection (Chinen et al., 2007; Thrasher et al., 2005).

Unfortunately, 4 patients from the French trial and 1 from the UK study also developed acute T cell lymphoblastic leukaemia (T-ALL) resulting from insertional transactivation of LMO2 proto-oncogene (4 out of 5 patients) and associated insertion/deletion/translocation/mutation of other genes namely *BMI1* (transcriptional control), *CCDN2* (cell-cycle protein) and *Notch-1* (T cell survival and proliferation). Three of the patients from the French trial and one from the UK trial entered remission following standard chemotherapy while one patient from the French trial died due to refractory leukaemia (Gaspar et al., 2011a; Hacein-Bey-Abina et al., 2003, 2008, 2010; Howe et al., 2008).

Preliminary results obtained from an on-going multi-centre (Europe & USA) trial initiated in 2010 and involving a total of 8 patients indicates similar clinical benefits and no adverse events although follow up is short. The vector used in this trial is based on a SIN γ-retrovirus in which the IL2RG gene is driven from an internal EF1α promoter (see Table 2 for summary).

### 2.3 CGD

Chronic granulomatous disorder (CGD) is a rare, inherited, autosomal recessive or X-linked disorder predominantly affecting neutrophil function. The condition arises from mutations affecting any one of the five genes encoding phagocytic oxidase (phox) proteins which form the subunits of the NADPH oxidase enzyme complex. In healthy subjects, phagocytic engulfment of microbes by neutrophils in the peripheral blood triggers the assembly of NADPH oxidase complex by translocation of cytosolic phox proteins (p47^phox^, p67^phox^ and p40^phox^) to the membrane bound flavocytochrome (gp91^phox^ and p22^phox^). Henceforth, the fully assembled NADPH oxidase complex catalyses the transfer of electrons from NADPH to molecular oxygen leading to a cascade of events involving rapid generation of superoxide anions, production of reactive oxygen species (ROS), and activation and release of antimicrobial proteases resulting in the efficient elimination of invading microbes. In contrast, CGD patients fail to generate this “respiratory burst” and display enhanced susceptibility to a wide-spectrum of bacterial and fungal infections, with the greatest threat of infection and mortality to X-linked CGD patients (two-thirds of CGD cases, gp91^phox^-deficient) (Jones et al., 2008; Segal, 2005; Segal et al., 2011; Winkelstein et al., 2000).

Other than life-long prophylactic treatment with antibiotics/antimycotics and recombinant interferon (IFN)-γ treatment, myeloablative allogeneic HSCT using a closely matched related or unrelated donor constitutes a definitive treatment option for children with CGD, although not without associated complications (Seger, 2008).

Being a monogenic disorder, the rationale for gene therapy of CGD patients lacking a HLA-matched donor is well-established on the following grounds—a) all the genes affecting NADPH oxidase subunits have been cloned, and b) data from healthy X-CGD carriers have revealed that significant functional correction of a minor fraction of neutrophils (approximately 10%) can benefit the patient considerably. However, CGD remains one of the most difficult targets for gene therapy for the following reasons:

a) Expression of the wild-type gene does not provide any survival advantage to the transduced stem and progenitor cells thereby necessitating the use of myeloablative conditioning to complement for this defect.
b) Unlike T cells, circulating neutrophils have a life-span of few days which means a large number of long-term repopulating HSCs need to be corrected for a positive clinical outcome.
c) An inflammatory environment in the bone marrow compartment could exert a negative effect on the successful engraftment of the transduced CD34^+^ cells (Aiuti et al., 2012; Grez et al., 2011).

The first two rounds of clinical trial conducted at NIH (USA) in 1995 and 1998 targeting autosomal recessive (p47^phox^ deficiency) and X-linked CGD (gp91^phox^ deficiency) with γ-retroviral vectors were performed on a total of ten patients without any pre-conditioning. In both the trials, trace numbers of oxidase-normal neutrophils persisted in the peripheral blood for a few months but failed to confer any long-term clinical benefit. Despite major increases in transduction rate (70% compared to 20%), number of cycles of gene therapy (4 compared to 1), and total number of gene-corrected CD34 + cells that were infused (10 × 10^6^/kg compared to 0.5 × 10^6^/kg), gene marking failed to surpass 0.13% at any peak time in the second trial. It is however noteworthy that individual gene-marked neutrophils in peripheral circulation displayed ability to produce physiologically normal levels of ROS upon stimulation (Goebel and Dinauer, 2003; Kang et al., 2010; Malech, 2000; Malech et al., 1997).

Results from these two US trials, and corresponding data from the SCID-X1 trials (where empty T cell compartment facilitates engraftment of gene-corrected progenitors) encouraged researchers from Germany and Switzerland to initiate ex-vivo gene therapy for X-CGD (4 patients in total; 2 young adults treated in the Frankfurt, and 2 children in Zurich) incorporating non-myeloablative conditioning and employing a γ-retroviral vector with a spleen focus forming virus (SFFV) derived LTR driving gp91^phox^ expression. Overall, within the first 5 months of transplantation, both patients in the Frankfurt trial demonstrated gene-marking in approximately 15% of circulating neutrophils with significant levels of ROS activity. Contrary to previous experiences, gene-marked neutrophils continued to increase to up to 50% of the total neutrophils in peripheral blood indicating lone-term engraftment and the patients were able to clear almost all pre-existing infections. However, the temporal increase in gene-marked neutrophils was found to be oligoclonal in nature caused by vector insertion into *MDS/EVI1* oncogene loci and their subsequent trans-activation by the SFFV LTR. Both patients developed myelodysplasia with monosomy 7 within the next 2–1/2 years, with corresponding loss of oxidase function in the gene marked cells, caused by methylation of the retroviral promoter. Unfortunately, one of the patients died from severe sepsis. Of the two children treated in Zurich, one developed MDS while the other displayed clonal expansion without MDS. Both patients have survived so far after HSCT (Bianchi et al., 2009, 2011; Ott et al., 2006; Stein et al., 2010).

In London, patients treated with the same vector achieved less than 10% gp91^phox^ positive neutrophils in peripheral blood at day 21 post-infusion with weak respiratory burst activity (5–10% of controls). By day 42, gene-marked cells became undetectable although without any SAE reported so far.

In a separate study at Seoul conducted on two patients with X-CGD, superoxide producing cells were detected in peripheral blood shortly after transplantation of gene-corrected CD34 + cells (mobilized from peripheral blood), but levels have dropped significantly (from a peak of 14.5% to 0.1%) afterwards in agreement with observations in other trials (Kang et al., 2011).

To summarize (Table 3), out of 13 X-CGD patients treated so far in combination with partial myeloablation, 10 patients have displayed only transient clinical benefit most likely due to low-level long term engraftment of transduced cells (Grez et al., 2011). Three patients displayed long-term efficacy but in all these cases clonal expansion was triggered by insertional activation of oncogene leading to SAE. Transgene silencing due to epigenetic modification was also witnessed in these patients.

### 2.4 WAS

Wiskott–Aldrich syndrome is a rare and severe X-linked immunodeficiency caused by mutations in the WAS gene encoding WAS protein (WASp), an actin cytoskeleton regulator expressed solely in cells of haematopoietic lineage. Mutations in WAS gene hamper cell migration, signalling and activation causing a host of defects leading to eczema, micro-thrombocytopenia, recurrent infections, autoimmunity and increased tendency to develop lymphomas. The overall incidence of classical WAS resulting from complete absence of WASp is estimated to be 4 per million live male births over a wide geographical area and amongst diverse ethnic groups. Severely reduced expression of WASp has been implicated in a milder phenotypic variant of WAS known as hereditary X-linked thrombocytopenia (XLT) or intermittent XLT. Intriguingly, a third allelic variant known as X-linked neutropenia (XLN) has been found to result from an extremely rare gain-of-function mutation. XLN does not present with any of the symptoms associated with WAS/XLT (Thrasher and Burns, 2010).

HSCT is recognized as a standard curative procedure for WAS although the success rate is determined by availability of HLA-matched donors and presence of a low clinical score at the time of treatment. For patients without suitable donors or at high risks of complications, WAS is an excellent candidate for gene therapy. Low levels of WASp expression in XLT patients have been found to confer considerable immunocompetence as well as survival advantage to mature lymphocytes. Together, these observations provide a strong rationale for WAS gene therapy since low level correction can be expected to provide considerable clinical benefit to the patient (Moratto et al., 2011).

Proof-of-concept studies in animal models have demonstrated both efficacy and feasibility of gene therapeutic approaches for treating WAS leading to the first clinical trial in which 10 patients were enrolled and treated with a GALV-pseudotyped γ-retroviral vector following busulfan conditioning. The initial reports for two patients, three years after gene therapy showed restored expression of WASp in multiple lineages, with increasing proportions of corrected lymphocytes over time confirming expected selective advantage. Restored gene expression correlated with improvements in platelet counts and corresponding resolution of bleeding, eczema and auto-immunity. Although the immunoglobulin levels did not rise to normal levels, both the patients were successfully vaccinated against tetanus, diphtheria and haemophilus influenza. Unfortunately, further reports confirmed the occurrence of leukaemia in 4 patients due to insertional transactivation of proto-oncogenes *MDS/EVI1* and *LMO2* by strong enhancer elements present within the viral LTR (Boztug et al., 2010).

A multi-centre trial has recently been initiated in the UK, the US, France and Italy. These trials will employ a VSV-G pseudotyped SIN lentivector driving expression of the WAS cDNA from an endogenous 1.6 kb long human WAS promoter. This international multicentre approach is expected to facilitate gathering of data (safety, multi-lineage reconstitution, clinical efficacy) across the board by maintaining uniform parameters of pre-conditioning and vector quality (Avedillo et al., 2011; Dupre et al., 2006; Galy et al., 2008; Scaramuzza et al., 2013) (see Table 4 for summary).

## 3 Pitfalls: safety issues and challenges

Irrespective of their successes or setbacks, all gene therapy clinical trials targeting the various forms of PIDs have imparted immensely valuable lessons to researchers on the various aspects of gene therapy. While the positive clinical outcome of ADA-SCID patients treated with gene therapy gave a major boost to the advancement of the field, the serious adverse events observed in the SCID-X1 trials led to the development of a nascent area of research (integration site analysis) focussed on vector-mediated oncogenesis. This contributed to improving safety standards for future gene therapy vectors. Again, the transient clinical benefit seen in CGD patients underlined the importance of pre-conditioning of the bone-marrow to maximise engraftment potential for the gene-corrected HSCs in cases where gene correction does not confer any selective advantage compared to untransduced HSCs.

It is still unclear which definite factors contribute to the risk of insertional mutagenesis associated with specific diseases and/or clinical trials. There are several possible explanations which are summarized in the following sections.

### 3.1 Nature of the target gene and disease compartment

ADA is a constitutively expressed metabolic enzyme while IL2RG is a component of a signalling complex that induces cell proliferation and is up-regulated upon T-cell activation. Therefore, it is conceivable (though not proven) that inappropriate expression of vector-mediated IL2RG at various stages of differentiation can be significantly detrimental. Similarly, it is possible that upon insertional activation, the cellular proto-oncogenes will interact with IL2RG expression in a synergistic fashion to cause tumorigenesis. The predisposition to lymphoma has been reported in two mouse models of SCID-X1 irrespective of γ-chain related oncogenicity. Therefore, both the nature of the gene product and the disease environment (cellular factors and replicative stress) might play a crucial role in determining the background risks associated with correcting the disease phenotype (Dave et al., 2009; Scobie et al., 2009; Shou et al., 2006).

### 3.2 Vector design and integration profile

The observed toxicities in the gene therapy clinical trials for SCID-X1, CGD and WAS share a common mechanism, namely up-regulated expression of proto-oncogenes induced by powerful enhancer sequences present in the LTRs of the early-generation γ-retroviral vectors (γ-RVs) used in majority of the clinical trials conducted so far. Integration site analysis of patient cells post gene therapy has revealed that these γ-retroviral vectors have a semi-random (25% of integrants in common insertion sites) yet enhanced tendency to target loci of actively transcribing genes during the time of transduction. Vectors have been found to integrate both within and near (promoter region) gene encoding proteins with kinase or transferase activity, or involved in phosphorus metabolism indicating that the survival potential of gene-corrected cells might be heavily dependent upon the site/s of integration. This in turn influences the ability of the transduced cells to engraft, differentiate and expand.

Enhancer-mediated up-regulation of *LMO2* proto-oncogene has been directly implicated in the leukemic outcomes of several SCID-X1 patients although the development of full-blown T-ALL might have been influenced by other genotoxic changes independent of retroviral vector integration. Similar observations have been made for the four WAS patients treated with gene therapy where T-cell leukaemia was associated with integrations in the vicinity of *LMO2* and other proto-oncogenes. Likewise, insertional transactivation of myeloproliferative genes like *MDS1/EVI1*, *PRDM16* or *SETBP1* has resulted in clonal dominance and malignant expansion in some CGD patients. In addition to pro-malignant tendencies, LTR-driven γ-RVs are also susceptible to epigenetic silencing by promoter methylation as was observed in the case of CGD patients (Cattoglio et al., 2010; Deichmann et al., 2011).

Intriguingly, although integration hotspots were identified in transcriptionally active regions close to proto-oncogenes in some ADA-SCID patients receiving gene therapy, clonal dysregulation has not been reported so far. It is possible that intrinsic metabolic or proliferative defects restrict leukemogenic potential in this case, as mutagenesis has been described in all other primary immunodeficiencies treated with LTR-based γ-RVs.

## 4 Advancements and prospects for PID gene therapy

### 4.1 Refining viral vector design

#### 4.1.1 Self-inactivating (SIN) vectors

The crux of novel viral vector development centres on elimination of endogenous enhancer elements responsible for insertional genotoxicity. Self-inactivating (SIN) γ-RVs have been incorporated in trans-Atlantic clinical studies in the recent past and the early results in SCID-X1 patients have been found to be encouraging. These vectors have been genetically engineered to carry a significant deletion within the U3 region (including CAAT and TATA boxes) of the viral LTRs of the integrated provirus thereby making them less likely to initiate any transcriptional activity of adjacent proto-oncogenes.

#### 4.1.2 Emergence of LVs

One paradigm shift in recent pre-clinical trials has been the employment of lentiviral vectors (LVs) over γ-RVs. Novel pre-clinical assays have shown that LVs have a decreased propensity to integrate near the regulatory elements of actively transcribed genes thereby significantly reducing the probability of insertional mutagenesis. Also, unlike γ-RVs, LVs are capable of transducing non-dividing cells thereby reducing the duration of ex-vivo culture of patient HSCs by eliminating the need for their treatment with cell-division inducing cytokines prior to transduction. Limited ex-vivo culture preserves the “stemness” of the multi-potent HSCs which is critical for successful long-term engraftment. Additionally LVs allow for inclusion of larger and more sophisticated transgene expression cassettes.

#### 4.1.3 Internal promoters, tissue-specificity and safety elements

SIN vectors typically express the gene-of-interest from an internal heterologous promoter such as SFFV, PGK or EF1α. These ubiquitously expressing promoters are capable of driving a strong and sustained expression of the transgene in various target cells but suffer from inherent disadvantages such as over-expression (above normal physiological levels) and mistimed expression (in early progenitors and precursors) on the one hand, and silencing of the promoter by methylation on the other (Kildebeck et al., 2012).

Incorporation of the ubiquitous chromatin opening element (UCOE) in the vector backbone has shown efficacy in resisting methylation of the heterologous promoters in pre-clinical animal/human xenograft models (Zhang et al., 2007). Similarly, introduction of endogenous (WAS gene promoter) and tissue-specific promoters (for example, CD19 for B cells, CD4/Lck for T cells, and C-Fes/Cathepsin G chimeric promoter for myeloid expression) is currently being investigated to restrict transgene expression in specific cell types and in response to physiological cues (Marodon et al., 2003; Moreau et al., 2004; Santilli et al., 2011). Limiting expression of transgene at the transcriptional level has also been achieved by incorporating microRNA (miR) target sequences in the therapeutic vectors which would ensure robust transgene expression in cells where the corresponding miR is absent. As an example, miR-126 is expressed in HSCs but not in differentiated blood cells. Addition of miR-126 target sequence in the vector backbone will therefore prevent expression of the transgene in the stem cells but support robust expression in relevant mature hematopoietic cells (Gentner et al., 2010).

An additional level of vector safety against position effect can be achieved by the introduction of chromatin insulator elements which define domains of transcriptional autonomy. One of the best characterized insulator elements is the chicken hypersensitive site-4(cHS-4) element of the beta-globin locus control region (β-LCR) which has both enhancer blocking and chromatin barrier effects.

### 4.2 Gene targeting

#### 4.2.1 Engineered endonucleases and homologous recombination

A promising alternative to gene-addition is in-situ replacement of disease-causing gene mutation by homologous recombination (HR) between the endogenous genomic sequence and an exogenous DNA template harbouring desired sequence alterations to correct the genetic defect. Correction of the diseased gene at its endogenous locus is expected to achieve therapeutic benefit without the accompanying complications of insertional oncogenesis, transgene silencing, and lack of gene regulation. HR strategy for gene correction requires double-strand breaks (DSBs) at the precise location of mutation. Engineered nucleases such as meganucleases (MNs), zinc-finger nucleases (ZFNs) and transcription activator-like effector nucleases (TALENs) have been designed to create such DSBs. In the context of PIDs, development of a IL2RG-specific ZFN pair targeting exon 5 (hotspot for SCID-X1 mutations) has been found to affect targeted gene replacement in both human cell lines and primary T cells with frequencies of 18% and 5% respectively (Lombardo et al., 2007; Urnov et al., 2005; Wood et al., 2011). Similar efficacy has been demonstrated in correcting gp91^phox^ mutation by ZFNs in human X-CGD patient-derived induced pluripotent stem cells (Zou et al., 2011). Unfortunately, both ZFNs and TALENs have been associated with genotoxic “off-target effects” caused by non-specific DSBs in the genome. Development and optimization of safe and effective engineered endonucleases for PIDs are areas of intense current research.

#### 4.2.2 Genomic safe harbours

Genomic safe harbours (GSHs) are regions of the human genome where newly integrated transgenes can be expressed stably without adverse effects on the host cell or organism (for example using various HR strategies). Various GSHs are currently being investigated and validated by laboratories around the world and few of the prime intragenic candidates are the AAVS1 site in chromosome 19, and the CCR5 and ROSA26 loci in chromosome 3. It is important to note that a fully defined GSH has not yet been identified (Sadelain et al., 2012).

## 5 Conclusions

The overall impressive efficacy of gene therapy for PIDs, when compared to that obtained by transplantation with non-HLA-identical HSCs, provides a strong argument in favour of continued exploration of this therapeutic approach. The strong rationale for PID gene therapy has been strengthened further by technological advancements in the design and manufacture of clinical grade GMP-quality vectors coupled with enhanced transduction protocols and improved transplantation regimens. SAEs constitute a prime concern in the clinical trials conducted so far targeting SCID-X1, CGD and WAS. Although the exact mechanism of malignant transformation observed in these trials remains unresolved, it is possible that multiple factors contribute to oncogenesis. It is worth mentioning here that allogeneic transplantation of HLA-mismatched HSCs also suffers from occurrences of life-threatening graft-versus-host-disease (GvHD). Encouraging results from the PID clinical trials (as described above) have set the platform for addressing other monogenic disorders (both haematopoietic and metabolic) with gene therapy, especially for those in which the transgene-expressing cells have an anticipated selective advantage over the cells harbouring the mutation.

In the past, pre-clinical study of various PIDs has been handicapped by the severely restricted availability of primary patient samples owing to their rare occurrence as well as the fact that many of the PIDs affect infants and young children. However, a significant biomedical breakthrough in the form of induced pluripotent stem cell (iPSC) technology promises to be a potent weapon in allowing patient-specific disease modelling for PIDs by overcoming the issue of limited availability of biopsy samples. Proof-of-concept studies have already been carried out both in humanized PID animal models and patient samples to confirm the utility of this technology in serving as a platform for optimization and in-depth study of gene correction (Pessach and Notarangelo, 2011; Pessach et al., 2011).

A key consideration in adopting gene therapy of PIDs as a viable treatment option is to develop a robust business model to balance profitability of gene therapy developers with affordability of treatments for patients. Although the present costs of treating PID patients with gene therapy remain substantially high (due to infrastructural requirements, GMP-quality vector manufacture, long-term follow-ups), it can be envisaged that for a large number of monogenic disorders, the one-and-done treatment solution offered by gene therapy will effectively counterbalance the chronic treatment costs incurred during the life-time of patients. A paradigm shift in therapeutic landscape of PIDs will also require coordination of resources and ideas between public and private enterprises to clearly evaluate the potent health economics argument in favour of gene therapy.

## Figures and Tables

**Table 1:** Summary of recent clinical trials targeting ADA-SCID. BM, bone marrow; UCB, umbilical cord blood; ERT, enzyme replacement therapy; SAE, serious adverse event.

Trial centre	Vector	Target	Conditioning	Patients	Outcome	SAE	References
Italy	γ-Retrovirus	BM CD34^+^	Busulfan 4 mg/kg	18	15/18 off ERT	None	Aiuti et al. (2009)
UK	γ-Retrovirus	BM CD34^+^	Melphalan 140 mg/m^2^ or Busulfan 4 mg/kg	8	4/8 off ERT	None	Gaspar et al. (2011b)
USA	γ-Retrovirus	UCB CD34^+^	Busulfan 4 mg/kg	14	10/14 off ERT	None	Candotti et al. (2012)
UK, USA	SIN lentivirus EF1α promoter		Busulfan 4 mg/kg	2	Follow-up less than a year		*Personal communication* Gaspar HB

**Table 2:** Summary of recent clinical trials targeting SCID-X1. T-ALL, T-cell acute lymphoblastic leukaemia bone marrow; LMO2, LIM domain only 2; SAE, serious adverse event.

Trial centre	Vector	Conditioning	Patients	Outcome	SAE	Insertion site(s)	References
France	γ-Retrovirus (amphotropic)	None	9	Significant clinical benefit to most	4 developed T-ALL; 1 died	LMO2 CCND2 BMI1	Hacein-Bey-Abina et al. (2002) Hacein-Bey-Abina et al. (2003) Hacein-Bey-Abina et al. (2008) Hacein-Bey-Abina et al. (2010)
			1	No clinical benefit (age related)			Thrasher et al. (2005)
UK	γ-Retrovirus (GALV)	None	10	Significant clinical benefit to most	1 developed T-ALL	LMO2	Gaspar et al. (2011a) Howe et al. (2008)
			1	No clinical benefit (age related)			Thrasher et al. (2005)
USA	γ-Retrovirus (GALV)	None	3	Limited clinical benefit (age-related)	None	–	Chinen et al. (2007)
France, UK, USA	SIN γ-retrovirus EF1α promoter	None	8	T-cell recovery observed (preliminary results)	–	–	*Personal communication* Thrasher AJ

**Table 3:** Summary of recent clinical trials targeting CGD. SAE, serious adverse event.

Trial centre	Vector	Conditioning	Patients	Outcome	SAE	Insertion site(s)	References
US	γ-Retroviral (amphotropic)	None	5	No clinical benefit	None	–	Malech et al. (1997) Malech (2000) Goebel and Dinauer (2003) Kang et al. (2010)
		None	5	No clinical benefit	None	–	
		Busulfan (10 mg/kg)	3	Transient clinical benefit	None	–	
Germany	γ-Retroviral (SFFV LTR)	Busulfan (8.8 mg/kg)	2	Long term clinical benefit	Both developed MDS with monosomy 7; 1 died from sepsis	MDS EVI1	Ott et al. (2006) Stein et al. (2010) Bianchi et al. (2009) Bianchi et al. (2011)
Switzerland			2	Transient clinical benefit	1 patient developed MDS	None	
UK	γ-Retroviral (MLV LTR)	Melphalan (140 mg/m^2^)	1	Transient clinical benefit	None	–	*Personal communication* Thrasher AJ
	γ-Retroviral (SFFV LTR)		3				
Korea	γ-Retroviral	Busulfan (6.4 mg/kg) + Fludarabine (120 mg/m^2^)	2	Transient clinical benefit	None	–	Kang et al. (2011)
Switzerland, Germany, France, UK	SIN lentivector, myeloid promoter	Busulfan (12–16 mg/kg)	1	Trial open	–	–	–

**Table 4:** Summary of recent clinical trials targeting WAS. SAE, serious adverse event; T-ALL, T cell acute lymphoblastic leukaemia.

Trial centre	Vector	Conditioning	Patients	Outcome	SAE	Insertion site(s)	References
Germany	γ-Retroviral (LTR driven, GALV pseudotyped)	Busulfan (8 mg/kg)	10	Long-term correction	T-ALL in 4/10 patients	LMO2 MDS/EVI1	Boztug et al. (2010)
UK, US, France, Italy	SIN lentivector (endogenous WAS promoter, VSV-G pseudotyped)	Busulfan + Fludarabine +/− ATG/Rituximab	5	Multilineage correction (preliminary results)	–	–	
